# Supplementary material for: Altered differentiation of endometrial mesenchymal stromal fibroblasts is associated with endometriosis susceptibility
Source: Commun Biol. 2022 Jun 20;5:600. doi: 10.1038/s42003-022-03541-3 (PMC9209414; doi:10.1038/s42003-022-03541-3)
Supplement: Supplementary file 2 — Supplementary information [file 42003_2022_3541_MOESM2_ESM.pdf]

Supplementary Table 1: Patient Characteristics

	**Age**	**p Value**	**BMI**	**p Value**
**Case**	35.17	0.939	24.16	*0.0039*
**Control**	35		27.29	

	Menstrual stage			
	**Proliferative**	**Periovulatory**	**Secretory**	**Stage p**
**Case**	7	1	2	0.116
**Control**	2	3	4	

	Passage Number				
	**4**	**5**	**6**	**7**	**p**
**Case**	2	0	7	1	*0.026*
**Control**	2	4	1	0	

Supplementary Table 2: Covariate analysis of patient characteristcs

COVARIATES	FIB r^2	MSC r^2
Passage	-0.024	0.115
Menstrual phase	0.03	0.134
AGE	0.03	-0.038
Lesion subtype	-0.027	0.028

Supplementary Table 3: Cluster cell number relationship to patient characteristics

	**Age**		**BMI**		**Passage**	
	r2	*p*	*r2*	*p*	*F*	*p*
Cluster 0	-0.2601	0.3306	-0.1948	0.4386	0.1906	0.9011
Cluster 1	-805	0.767	0.3661	0.1352	0.4724	0.706
Cluster 2	0.0496	0.8552	-0.0065	0.9794	2.118	0.1408

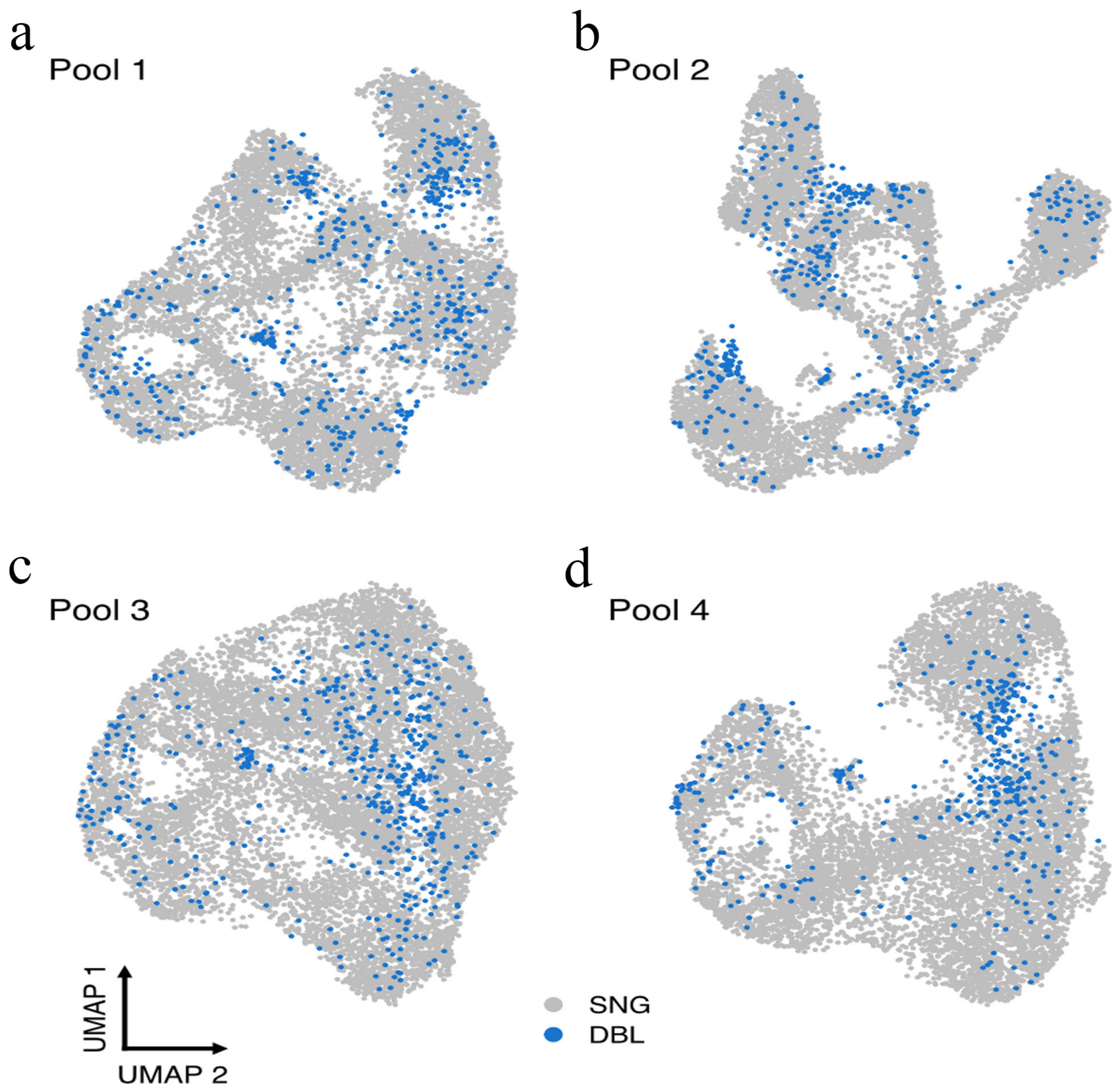

Supplementary Figure 1: Identification of singlets and doublets in scRNA-seq data. UMAP plots of all cells in the scRNA-seq experiment coloured by singlet and doublet status as determined via Demuxlet. Doublets were randomly spread between each pool. **(a)** Pool 1, **(b)** Pool 2, **(c)** Pool 3 and **(d)** Pool 4. Doublets were subsequently removed from downstream analysis. SNG = Singlet; DBL = Doublets.

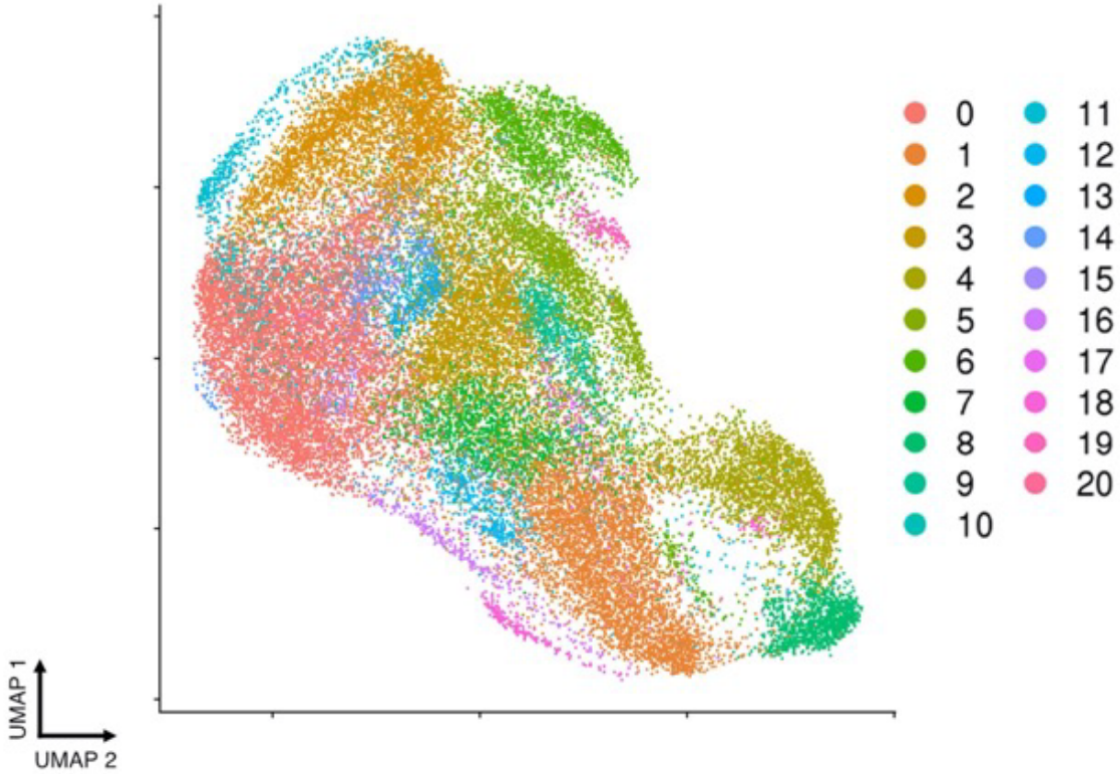

Supplementary Figure 2: Bioinformatic clustering of mesenchymal derived cells at increasingly finer resolution. Using the *clustree* software cell clusters can be defined at various levels of resolution. We examined the clusters created when resolution was defined at various levels, subsequently selecting a 0.6 resolution for subsequent analysis. Additional resolutions at 1.0 shows the increasing number of clusters that can be identified as resolution is increased.

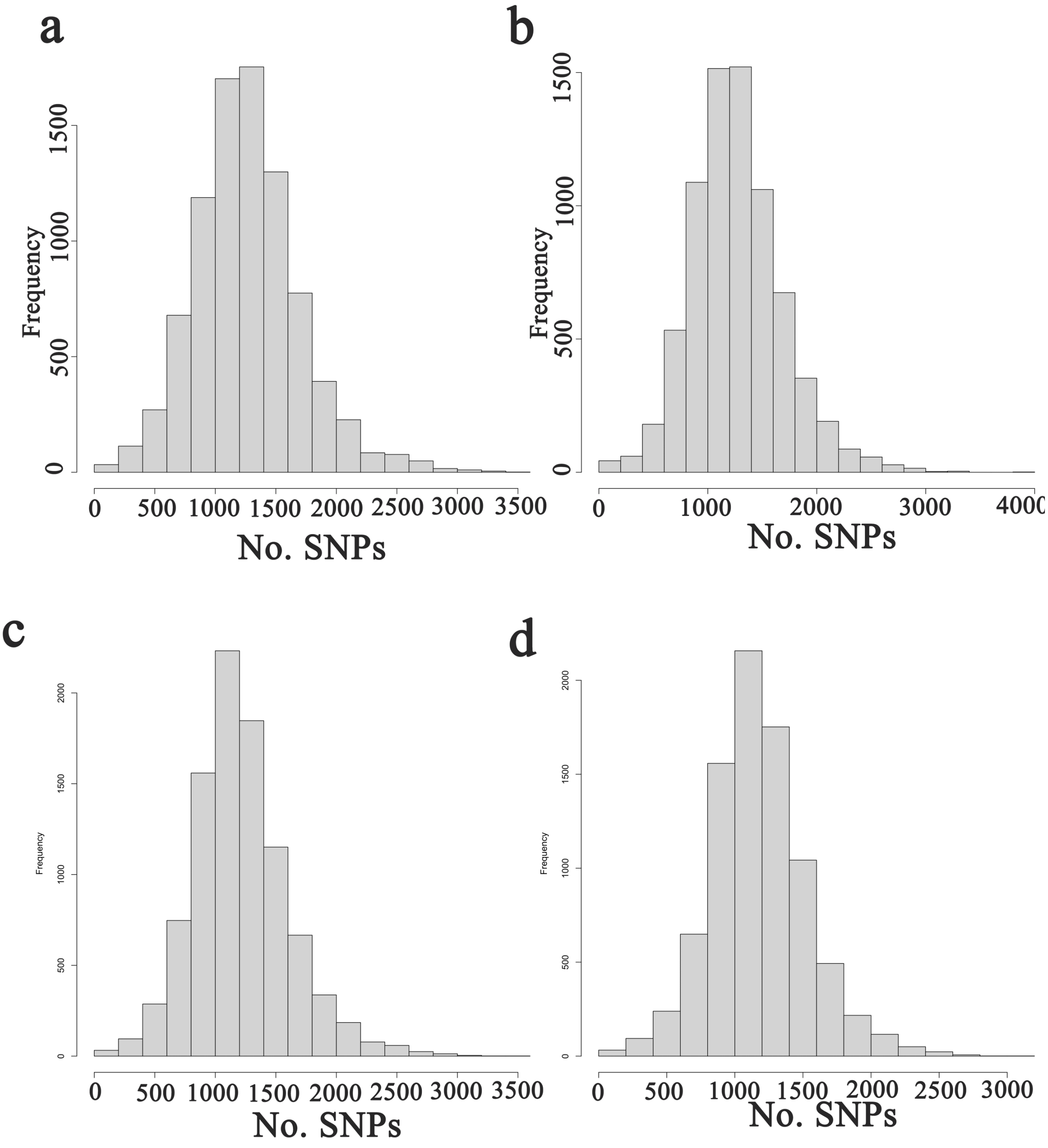

Supplementary Figure 3: Using genotyping information to identify individuals in each pool. Using genotype data generated from GSA chips SNPs were aligned against the scRNA-seq data using the Demuxlet software. The number of SNPs ranged from 13 – 3,970 SNPs/cell with the average number being **(a)** 1,264 SNPs/cell for pool 1, **(b)** 1,270 SNPs/cell for pool 2, **(c)** 1,213 SNPs/cell for pool 3 and **(d)** 1,176 SNPs/cell for pool 4.

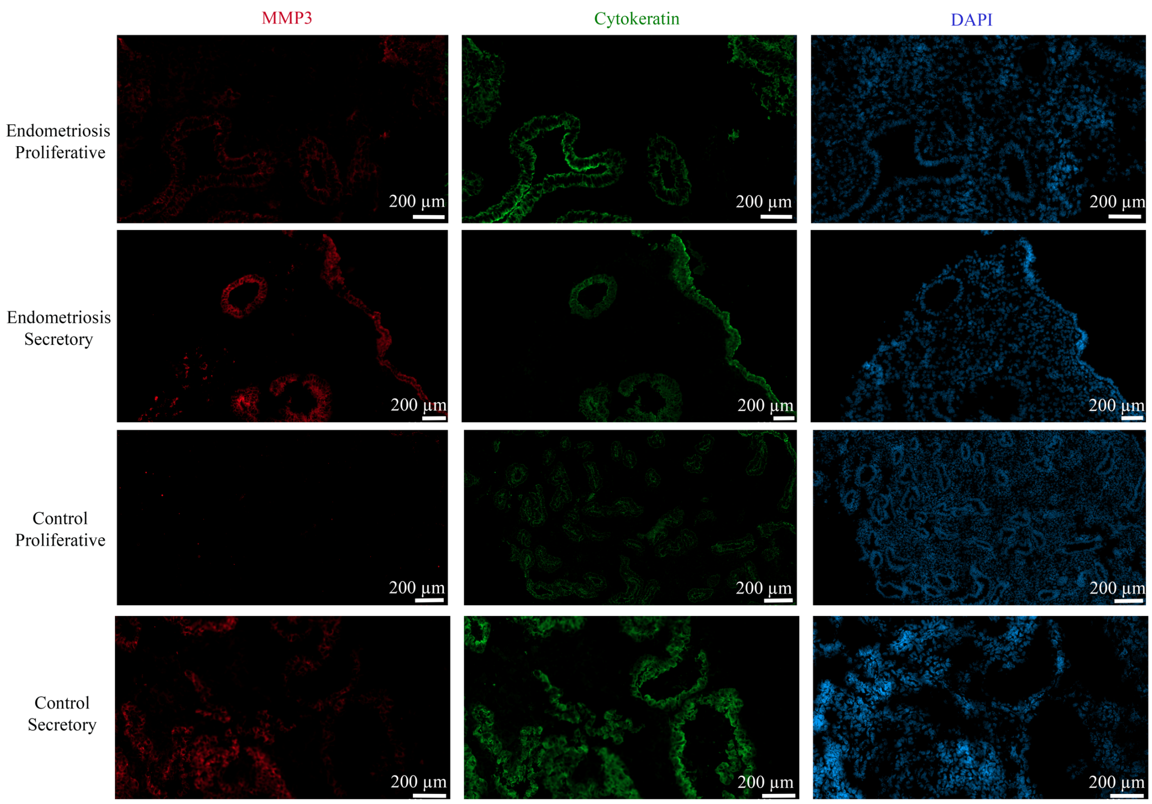

Supplementary Figure 4: Expression of MMP3 in endometrial tissue from women with and without endometriosis. MMP3 protein expression was determined by incubation of endometrial samples of women with and without endometriosis and from the proliferative and secretory stage of the menstrual cycle with specific antibodies for MMP3 (*red*) and Cytokeratin (*Green*). DAPI (*blue*) was used as a identify cell nuclei. Each row represent each individual channel used to generated figure 6 prior to merging into a single, multi colour image.

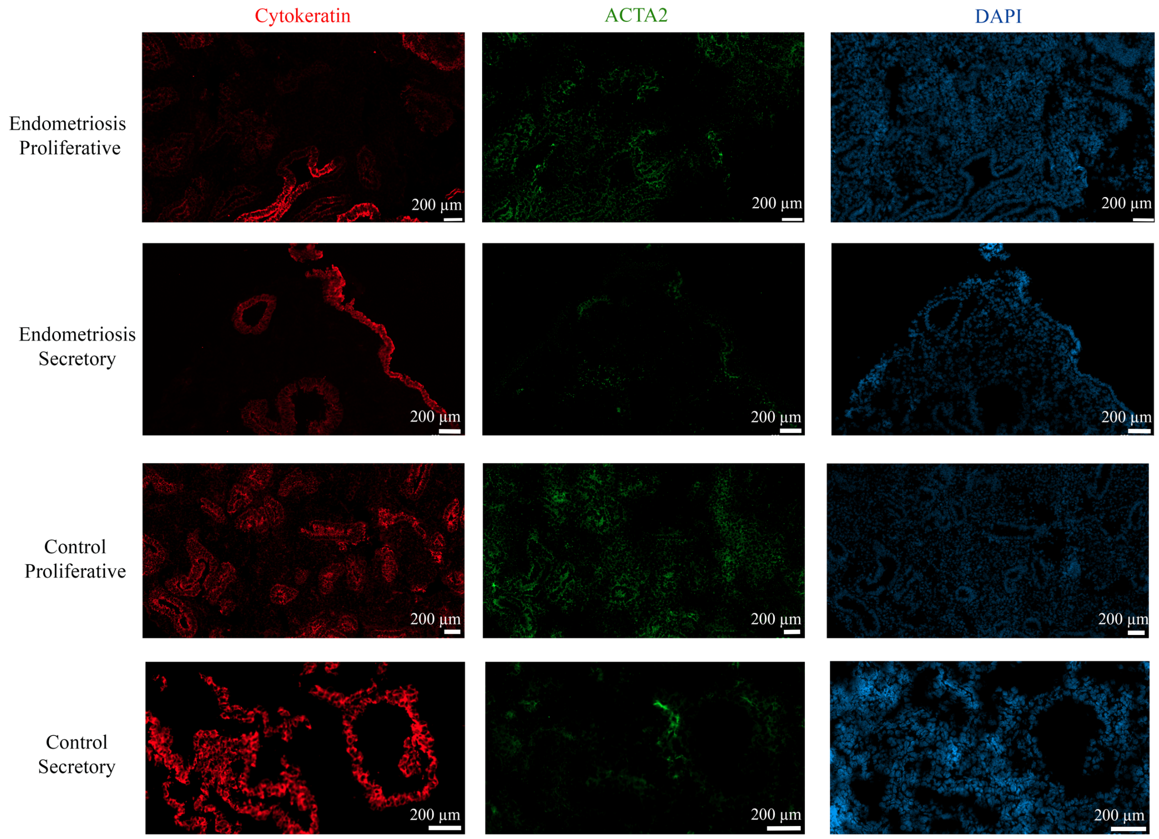

Supplementary Figure 5: Expression of ACTA2 in endometrial tissue from women with and without endometriosis. ACTA2 protein expression was determined by incubation of endometrial samples from women with and without endometriosis and from the proliferative and secretory stage were incubated with specific antibodies for ACTA2 (*green*) and cytokeratin (*Red*). DAPI (*Blue*) was used to identify cell nuclei. Each row represent each individual channel used to generated figure 6 prior to merging into a single, multi colour image.
