# Supplementary material for: Altered differentiation of endometrial mesenchymal stromal fibroblasts is associated with endometriosis susceptibility
Source: Commun Biol. 2022 Jun 20;5:600. doi: 10.1038/s42003-022-03541-3 (PMC9209414; doi:10.1038/s42003-022-03541-3)
Supplement: Supplementary file 3 — Description of Additional Supplementary Files [file 42003_2022_3541_MOESM3_ESM.pdf]

Description of Additional Supplementary Files

File name: Supplementary Data 1
Description: Differential gene expression analysis between Cluster 0 and all other cells.

File name: Supplementary Data 2
Description: Differential gene expression analysis between Cluster 1 and all other cells.

File name: Supplementary Data 3
Description: Differential gene expression analysis in cells from cluster 2 to all other cells.

File name: Supplementary Data 4
Description: Significantly different gene expression between cluster 0 and cluster 1.

File name: Supplementary Data 5
Description: Significantly different gene expression between cluster 1 and cluster 2.

File name: Supplementary Data 6
Description: Significantly different gene expression between cluster 0 and cluster 2.
