# Supplementary material for: Altered differentiation of endometrial mesenchymal stromal fibroblasts is associated with endometriosis susceptibility
Source: Commun Biol. 2022 Jun 20;5:600. doi: 10.1038/s42003-022-03541-3 (PMC9209414; doi:10.1038/s42003-022-03541-3)
Supplement: Supplementary file 10 — Reporting Summary [file 42003_2022_3541_MOESM10_ESM.pdf]

nature portfolio

Corresponding author(s): DBPR COMMSBIO-21-1458B

Last updated by author(s): Apr 19, 2022

Reporting Summary

Nature Portfolio wishes to improve the reproducibility of the work that we publish. This form provides structure for consistency and transparency in reporting. For further information on Nature Portfolio policies, see our Editorial Policies and the Editorial Policy Checklist.

Statistics

For all statistical analyses, confirm that the following items are present in the figure legend, table legend, main text, or Methods section.

n/a	Confirmed	
☐	☒	The exact sample size (n) for each experimental group/condition, given as a discrete number and unit of measurement
☐	☒	A statement on whether measurements were taken from distinct samples or whether the same sample was measured repeatedly
☐	☒	The statistical test(s) used AND whether they are one- or two-sided<br>*Only common tests should be described solely by name; describe more complex techniques in the Methods section.*
☐	☒	A description of all covariates tested
☐	☒	A description of any assumptions or corrections, such as tests of normality and adjustment for multiple comparisons
☐	☒	A full description of the statistical parameters including central tendency (e.g. means) or other basic estimates (e.g. regression coefficient) AND variation (e.g. standard deviation) or associated estimates of uncertainty (e.g. confidence intervals)
☐	☒	For null hypothesis testing, the test statistic (e.g. F, t, r) with confidence intervals, effect sizes, degrees of freedom and P value noted<br>*Give P values as exact values whenever suitable.*
☒	☐	For Bayesian analysis, information on the choice of priors and Markov chain Monte Carlo settings
☒	☐	For hierarchical and complex designs, identification of the appropriate level for tests and full reporting of outcomes
☐	☒	Estimates of effect sizes (e.g. Cohen's d, Pearson's r), indicating how they were calculated

Our web collection on statistics for biologists contains articles on many of the points above.

Software and code

Policy information about availability of computer code

Data collection	All computer code used during this study is freely available through open source software
Data analysis	All computer code used during this analysis is freely available through open source software

For manuscripts utilizing custom algorithms or software that are central to the research but not yet described in published literature, software must be made available to editors and reviewers. We strongly encourage code deposition in a community repository (e.g. GitHub). See the Nature Portfolio guidelines for submitting code & software for further information.

Data

Policy information about availability of data

All manuscripts must include a data availability statement. This statement should provide the following information, where applicable:

- Accession codes, unique identifiers, or web links for publicly available datasets
- A description of any restrictions on data availability
- For clinical datasets or third party data, please ensure that the statement adheres to our policy

In accordance with our HREC approvals at The University of Queensland and Bern Canton Ethics Committee and the open source policy for our funding for this study, data can be made available upon reasonable request to the corresponding author

Field-specific reporting

Please select the one below that is the best fit for your research. If you are not sure, read the appropriate sections before making your selection.

☒ Life sciences ☐ Behavioural & social sciences ☐ Ecological, evolutionary & environmental sciences

For a reference copy of the document with all sections, see nature.com/documents/nr-reporting-summary-flat.pdf

Life sciences study design

All studies must disclose on these points even when the disclosure is negative.

Sample size	Sample sizes were chosen to include as large a sample size of distinct patients that would be achievable within our budget.
Data exclusions	No data has been excluded from the analysis
Replication	Where possible experiments have been replicated by performing on multiple patient samples. All replicates have been reported
Randomization	Samples were allocated to experimental groups based on their endometriosis status. For all experimental procedures most experiments were performed in a single batch with all samples run simultaneously. If this was not possible it has been mentioned in the manuscript and these groups were randomized in any such experiments .
Blinding	Researchers performing the data analysis were blinded to any data associated with the individual samples.

Reporting for specific materials, systems and methods

We require information from authors about some types of materials, experimental systems and methods used in many studies. Here, indicate whether each material, system or method listed is relevant to your study. If you are not sure if a list item applies to your research, read the appropriate section before selecting a response.

Materials & experimental systems

n/a	Involved in the study	
☐	☒	Antibodies
☒	☐	Eukaryotic cell lines
☒	☐	Palaeontology and archaeology
☒	☐	Animals and other organisms
☐	☒	Human research participants
☒	☐	Clinical data
☒	☐	Dual use research of concern

Methods

n/a	Involved in the study	
☒	☐	ChIP-seq
☐	☒	Flow cytometry
☒	☐	MRI-based neuroimaging

Antibodies

Antibodies used	Cytokeratin Mouse Novus Biotechnie Cat No: NBP2-29429, Cytokeratin Rabbit Abcam (Cat No:Ab9377), MMP3 Rabbit Abcam (Cat No: AB52915), ACTA2 Mouse Thermo Fischer (Cat No: 14-9760-82)
Validation	Validation of antibodies was based on manufacturers reporting and specificity identify during in house staining procedures.

Human research participants

Policy information about studies involving human research participants

Population characteristics	All covariate information for the studied sample collection is included in the Manuscript in Supplementary Table 1
Recruitment	Patients were recruited as part of an ongoing study that include all patients alreadu undergoing surgery for endometriosis or suspected endometriosis
Ethics oversight	Tissue sample collection was approved by the Cantonal ethics commission Bern (149/03) and the Metro North Human Research Ethics committee (2019/QRBW/56763). Experimental procedures were approved by the Cantonal ethics commission Bern (2019-01146) and the University of Queensland Human Research ethics committee (2016001723) (2019/HE002744).

Note that full information on the approval of the study protocol must also be provided in the manuscript.

Flow Cytometry

Plots

Confirm that:

☒ The axis labels state the marker and fluorochrome used (e.g. CD4-FITC).

☒ The axis scales are clearly visible. Include numbers along axes only for bottom left plot of group (a 'group' is an analysis of identical markers).

☒ All plots are contour plots with outliers or pseudocolor plots.

☐ A numerical value for number of cells or percentage (with statistics) is provided.

Methodology

Sample preparation	Complete sample preparation is provided in the manuscript
Instrument	Aria II FACS machine (Becton Dickinson)
Software	Built in software in the Aria II FACS was used. No specific analysis of FACS data was performed
Cell population abundance	Cell abundance is described in the manuscript and was subsequently used for single cells sequencing, providing in depth information bout the cell content
Gating strategy	Gating stratergies have been detailed in the manuscript. We used FACS only to determine cell purity

☒ Tick this box to confirm that a figure exemplifying the gating strategy is provided in the Supplementary Information.
